# Surveillance for Asiatic filoviruses and zoonotic paramyxoviruses at a perceived wildlife–human interface

**DOI:** 10.1080/22221751.2026.2713324

**Published:** 2026-08-27

**Authors:** Spencer L. Sterling, Neil Mittal, Benjamin Greene, Khwankamon Rattanatumhi, Sasiprapa Ninwattana, Mckenna D. Roe, Patarapol Maneeorn, Prateep Duengkae, Supaporn Wacharapluesadee, Eric D. Laing, Opass Putcharoen

**Affiliations:** a International Graduate Program in Medical Sciences, Faculty of Medicine, Chulalongkorn University, Bangkok, Thailand; b Department of Microbiology and Immunology, Uniformed Services University, Bethesda, MD, USA; c Thai Red Cross Emerging Infectious Diseases Clinical Center, King Chulalongkorn Memorial Hospital, Bangkok, Thailand; d Henry Jackson Foundation for the Advancement of Military Medicine, Bethesda, MD, USA; e Department of National Parks, Wildlife and Plant Conservation, Ministry of Natural Resources and Environment, Bangkok, Thailand; f Department of Forest Biology, Faculty of Forestry, Kasetsart University, Bangkok, Thailand

**Keywords:** Henipaviruses, parahenipaviruses, orthoebolaviruses, insectivorous bats, guano farming, wildlife-pathogen interactions, wildlife-pathogen-human interfaces

## Abstract

Emerging zoonotic viruses pose a global health threat. In Thailand, evaluating human-wildlife interfaces is critical for identifying potential zoonotic virus spillover, particularly in areas with high-risk contact with roosting bats. We conducted cross-sectional sampling of humans (n = 234) and bats (n = 272) at a cave site in central Thailand where guano farmers, national park workers, and cave visitors may be exposed to bat-borne viruses. Molecular and multiplex serologic methods were used to identify current and previous infections with zoonotic paramyxoviruses and filoviruses, while multivariable regression was used to identify human behavioral correlates of seroreactivity. We detected serological evidence of orthoebolaviruses in two cave-roosting bat species, *Mops plicatus* and *Hipposideros larvatus*, and evidence of exposure to small terrestrial mammal-borne parahenipaviruses in humans. Individuals working in the extractive industry were more likely to be seroreactive to parahenipaviruses, including Gamak virus (OR = 3.41, 95% CI: 1.39-8.57, P < 0.001). Parahenipaviruses have been detected in shrews in eastern China and South Korea; therefore, we used ecological niche modeling of *Crocidura* shrews to predict the potential host range of novel parahenipaviruses in Thailand. Four *Crocidura* species were predicted to occur in the study province. This study provides the first evidence of Asiatic filoviruses in bats in Thailand and human exposure to parahenipaviruses in Southeast Asia. These findings underscore the importance of zoonotic disease surveillance and support expanding sampling across animal species and serial time points to maximize detection of emerging zoonotic viruses and improve understanding of transmission dynamics at high-risk human-wildlife interfaces across ecological settings throughout Thailand and Southeast Asia.

## Introduction

Evidence indicates that bats (Order *Chiroptera*) are wildlife hosts to diverse viruses, including several that are highly virulent to livestock and humans [1]. However, *Chiroptera* is a diverse Order, and neither the virus-host status of each species or their associated viromes are fully characterized. For example, the limestone karst caves of Southeast Asia are roosting sites for many species of insectivorous bats, including free-tailed bats (genus *Mops*), horseshoe bats (genus *Rhinolophus*), and roundleaf bats (genus *Hipposideros*), numbering 1.5-2.5 million. Of these three bat genera, only horseshoe bats have been implicated as hosts of emerging viruses, such as sarbecoviruses, in Southeast Asia [2]. Bat guano farming is economically important among communities with access to caves across Southeast Asia, including Thailand, providing a profitable resource that can enhance local agricultural industries [3]. Whether the occupation of guano farming increases human vulnerability to bat-borne virus spillover remains unclear, though evidence suggests exposure to zoonotic diseases is associated with employment in extractive industries [4,5].

Alongside the aforementioned coronaviruses, viruses in the families *Paramyxoviridae* and *Filoviridae* have been designated as priority pathogens, and several are associated with bats as natural hosts including prototypic viruses such as Nipah virus (NiV) and Ebola virus (EBOV) [6,7]. NiV is a member of the genus *Henipavirus* and is found in tree-roosting flying fox populations across South and Southeast Asia. Interestingly, newly identified paramxyoviruses, such as Langya virus (LayV), which was discovered after an outbreak of acute respiratory illness in eastern China, have been found to circulate in populations of shrews in east China and South Korea [8,9]. In contrast to the henipaviruses, which utilize host ephrin-B ligands as virus receptors, the cellular receptor for these newly-described parahenipaviruses remains a mystery [5]. Asiatic filoviruses such as Reston virus (RESTV), Měnglà virus (MLAV) and Dehong virus (DEHV), have been detected bats species in the Philippines and south China, but their biothreat remains largely speculative [10,11]. No studies to-date in Thailand have detected either molecular or serologic evidence of filoviruses in any bat species [12].

Understanding the specific host–pathogen relationships in the cave systems where guano harvesting practices occurs is critical to developing targeted surveillance of wildlife-pathogen interactions. Therefore, identifying pathogen interfaces and wildlife-human interactions that increase the spillover risk of zoonotic viruses are key first steps in preventing zoonosis. Notably, certain occupations are perceived to increase the vulnerability of individuals and humans to zoonosis [13], but robust studies to empirically confirm those predictions are often lacking. Furthermore, just as wildlife-pathogen interactions are specific, so are the wildlife-pathogen-human interfaces. Thus, occupational risk may not be generalizable for all emerging zoonotic viruses, and effective evidenced-based intervention strategies need to be appropriately designed for each pathogen and interface. Furthermore, cryptic spillover events, or the spillover of disease that does not clinically overt disease or widespread outbreaks, are implied as a possibility in current zoonotic spillover models, necessitating investigations that may capture infectious agents not directly included in targeted assays [14]. Previous One Health-based biosurveillance efforts have identified cryptic spillover of zoonotic and human diseases among wildlife [15,16].

We previously examined the presence of coronaviruses at a perceived wildlife-human interface involving guano farmers and individuals who work in the national parks system and regulate cave tourism [17]. To characterize this perceived interface as a jump zone for bat-borne paramyxoviruses and filoviruses, we performed a series of cross-section sampling events in the human and bat populations and used molecular and multiplex serological assays to detect evidence of viral infection. We hypothesized that (1) despite no previous evidence of their presence within this population, bat-borne paramyxoviruses and filoviruses would be identified in the bat populations; and (2) occupational hazards such as frequency and contact with bats would increase the likelihood of having detectable infection histories with identified bat-borne paramyxoviruses and filoviruses.

## Methods

### Study site

The study proceedings occurred in Tambon Tao Pun, Ratchaburi, Thailand, where a karst cave complex hosts an estimated 1.5-2.5 million bats (order *Chiroptera*) including *Rhinolophus*, *Hipposideros*, and *Mops* species [18]. This site is integral to the cultural and economic systems of the immediate area: the Wat Kho Chon Pran temple complex is situated immediately outside of the cave entrance, and the caves serve as a collection area for the village’s guano extraction industry. Many members of the community are employed within this industry and participate in the weekly collection, processing, and packaging of bat guano [17]. The cave system and fauna additionally serve as a tourist attraction through the viewing of nightly flyouts. Additionally, a wildlife protective zone administered and staffed by the Thai Department of National Parks, Wildlife, and Plant Conservation (DNP) is located next to the village, and DNP staff routinely encounter regional wildlife and are often required to handle bats. This site has served as a target for One Health-based surveillance activities due to the perceived risk of zoonosis from the potentially large human-animal interface [17,19].

### Study participants

Sampling of human study participants occurred between 2017–2018 during the United States Agency for International Development Emerging Pandemic Threats PREDICT project (Chulalongkorn University IRB #380/59), and 2021–2023 through the US NIH Centers for Research in Emerging Infectious Diseases (CREID) project (Chulalongkorn University IRB# 211/64). Study participants were recruited after initial site visits and community members at or above the age of 12 were encouraged to enroll, especially if they or members of their household were employed in the guano collection industry or had close contact with wildlife. Individuals under 11 and non-residents were excluded from the study. Enrollment and sampling occurred in April 2017, June 2018, November 2021, and and January 2023. Participants were asked to complete an epidemiological questionnaire and were sampled for blood serum and either oral, nasal, or nasopharyngeal swab samples. Swab samples were immediately placed in 1 mL lysis buffer (BioMerieux, SA, March l’Etoile, France) containing guanine thiocyanate in 2 mL screw-top tubes. A maximum of 5 mL of blood was collected in 10 mL blood collection tubes, allowed to clot, then centrifuged at 3000 x G for 10 min. Serum was decanted and thermally inactivated at 56°C for 30 min. All samples were immediately stored on ice after processing and then transported for long-term storage at −80°C.

### Animal sampling

Animal sampling occurred in September, 2020 and October, 2021 through a CREID project (Chulalongkorn University IACUC 015/2556). Bats were captured over two nights each sampling event at their roost or feeding sites during their nightly fly out using mist nets and individually placed in cotton bags prior to non-lethal sampling as described previously [20]. Each bat was manually restrained and speciated based on morphological characteristics, then two rectal swabs were collected from each bat using sterile polyester-tipped swabs and stored in 1 mL lysis buffer containing guanidine thiocyanate in 2 mL screw-top tubes. Blood samples were collected at a maximum of 6 µL of blood per gram of bodyweight for bats under 100 grams or no more than 1% of blood-by-total bodyweight for bats above 100 grams. Blood was collected in EDTA capillary tubes, centrifuged, heat inactivated, and plasma was decanted and aliquoted as described above. After processing samples were stored on ice, then transferred to long-term storage at −80°C prior to laboratory activities.

### Nucleic acid extraction and complementary DNA (cDNA) synthesis

Nucleic acid was extracted from each human and bat swab sample using the MagDEA Dx SV automated extraction system (Precision System Science, Chiba, Japan) and the accompanying magLEAD Consumable Kit using the 200 µL elution protocol under enhanced BSL-2 conditions as per manifacturer’s recommendations. Eluted nucleic acid was then immediately processed for cDNA synthesis using the Superscript III First-Strand Synthesis System (Thermo Fisher, Waltham, MA, USA) under the manufacturers recommendations.

### Molecular screening: reverse transcription-polymerase chain reaction (PCR)

The reverse transcription-PCR methods and consensus PCR primers described in Zhai, et al. (2007), were used for the detection of filovirus nucleic acid [21]. Briefly, 5 µL of cDNA was combined with filovirus primers and the PCR master mix and incubated 94°C for 30 sec, 58° C for 50 sec, and 73°C for 90 sec for 35 cycles, then a seven-minute final extension. Samples were analysed via gel electrophoresis using a 2% agarose gel.

A nested PCR protocol described in Tong, et al. (2008), was used for henipavirus and parahenipavirus nucleic acid detection [22]. Briefly, 2 µL of internal control RNA was added to 5 µL of cDNA, nucleoprotein-specific primers, and the One Step RT–PCR kit (Qiagen Inc., Valencia, CA, USA) as previously described and incubated at the following conditions: 48°C for 30 min for reverse transcription, 94°C for 2 min for reverse transcription inhibition, then 40 cycles of 94°C for 15 s for denaturation, 49°C for 30 s for primer annealing, and 72°C for 30 s for elongation, and a final 72°C for 7 min. Samples were analysed via gel electrophoresis using a 2% agarose gel.

DNA products from both PCR reactions were analysed on the QIAxcel Connect system (Qiagen, Hilden, Germany) using the QIAxcel DNA Screening Kit (Qiagen). PCR products were aliquoted in 10 µL volumes and analysed under the HIGH-Sensitivity process profile. Based on the consensus PCR and nested PCR filovirus amplicons were considered positive with a band length of 593 bp, and henipavirus amplicons were considered positive with a 323 bp control band and 227 bp sample band length, respectively.

### Serological screening

Blood serum and plasma were tested for virus-reactive IgG antibodies with a multiplex microsphere-based immunoassay as previously described [14,23]. Briefly, soluble, trimeric native-like attachment glycoprotein ectodomains (GP) of Ebola virus (EBOV, Accession: NC_002549.1), Bundibugyo virus (BDBV, NC_014373.1), Bombali virus (BOMV, NC_039345.1), Sudan virus (SUDV, NC_006432.1), Taï Forest virus (TAFV, NC_014372.1), Reston virus (RESTV, NC_004161.1), Ravn virus (RAVV, EU500826.1), and Měnglà virus (MLAV, NC_055510.1); and tetrameric receptor-binding glycoproteins (G) of Hendra virus (HeV, NC_001906.3), Nipah virus-Bangladesh strain (NiV, PX984254.1), Cedar virus (CedV, NC_025351.1), Ghana virus (GhV, NC_025256.1), Mòjiāng virus (MojV, NC_025352.1), Langya virus (LayV, OM101125.1) and Gamak virus (GakV, MZ574407.1) were expressed in HEK293F cell culture systems and purified by size-exclusion and affinity chromatography [24,25]. Cell culture supernatant was collected from a stable HEK293F cell line transfected with an empty pcDNA3.1 expression vector, and purified along with the GP and G antigens to generate a mock antigen control. All purified GP, G, and mock antigens were covalently coupled to unique magnetic microspheres per the manufacturer’s instructions (Luminex Corporation, Austin, TX, USA). Protein availability at the time of testing resulted in LayV G testing being limited to human blood specimens, and TAFV GP testing limited to only bat blood specimens. All serum or blood samples were inactivated, diluted 1:400 in 1xPBS, then probed for binding-IgG as described previously [23,26]. G and GP-binding IgG were quantified using a BioPlex 200 HTF multiplexing system (BioRad, Hercules, CA, USA) measuring at least 100 microspheres for each bead region under high RP1 conditions and were reported as a median fluorescence intensity (MFI).

### Statistical analysis of serological data

All statistical analyses of antigen–antibody levels measured as MFI were performed in the statistical software *R* v4.5.1 [27] using the packages *stats* v3.6.2 (2019), *factoextra* v1.0.7 (2020), *mixtools* v2.0 (2022), *carat* v2.2.1 (2023), *glmnet* v4.10 (2025), and *boot* v1.32 (2025). Data was visualized using the package *ggplot2* v3.5.2 (2025). Serological profiling, including dimensionality reduction through principal component analysis (PCA), k-medoids clustering, and the generation of seroreactivity cutoffs using unsupervised univariate mixture models has been described previously [14,23]. Briefly, binding results were subset by mammalian genus and viral family, and PCA with k-medoids clustering was used to select specific viral species within each mammalian genus with demonstrable seroreactivity. Binding data for these viral species were subsequently selected for mixture model analysis to establish viral- and mammalian order- specific seroreactivity cutoffs. Subsequent generalized linear model Spearman’s correlation and a One-way ANOVA with subsequent Tukey’s analysis was calculated for MFI values from seroreactive samples using GraphPad Prism v10.6.1 (GraphPad Software, Boston, MA, USA).

### Epidemiological analysis

The questionnaire collected from human study participants consisted of 57 questions and an additional bat guano collection specific questionnaire consisting of 16 questions [28]. Questionnaire data were analysed at the individual participant level. For participants who were enrolled at multiple timepoints, questionnaire data collected in the year when samples were positive for nucleic acid or serological reactivity was used for analysis; when a participant was positive for multiple years the questionnaire data from the year with the most recent positivity was analysed; and questionnaire data from the most recent year of sampling was used for individuals with negative results from all collection points. Epidemiological data, including age group, gender, primary livelihood, and animal contact including contacts with bats, non-human primates, and rodents and the individual’s (1) bat-borne henipavirus, (2) small-mammal-borne henipavirus, and (3) filovirus serostatus was analysed using least absolute shrinkage and selection operator (LASSO) regression with subsequent post-selection Generalized Linear Modelling (GLM) to determine the key epidemiological factors associated with seroreactivity relative to individuals determined as seronegative. This regression model was chosen due to the quantity of predictor variables within our dataset and biases the resulting regression coefficients towards zero to avoid overfitting the resulting model [29]. Subsequent regression modelling to control for LASSO-based selection bias was performed using debiased LASSO regression, Elastic Net regression, and backwards stepwise logistic regression [30,31]. All levels of statistical significance were reported at an α = 0.05.

### Ecological niche modelling

Shrews were not sampled as part of the study design but still warranted exploration and evaluation for the potential of a terrestrial small mammal-human interface for spillover of parahenipaviruses. Ecological niche models (ENM) projections were created using *R* v4.5.1 (2025), *Maxent* v3.4.4 (2020) and *Java* v1.8.0_451 (2025) [32,33]. Additional packages used in R for data processing and display include *tidyverse* v2.0.0 (2023), *dismo* v1.3.16 (2024), *raster* v3.6.32 (2025), *rJava v1.0.11 (202*4), *terra* v1.8.60 (2025), *tidyterra* v0.7.2 (2025), *sf* v1.0.21 (2025), *rnaturalearth* v1.1.0 (2025), *rnaturalearthdata* v1.0.0 (2024), *geodata* v0.6-2 (2024), and *RColorBrewer* v1.1.3 (2022)*.*

Presence data in Asia for *Crocidura* shrews was obtained from the Global Biodiversity Information Facility (accessed September 8, 2025) [34]. Only occurrences observed after 1970 and derived from preserved specimens were selected. The WorldClim 2.1 dataset (accessed June 16, 2025) was utilized for environmental covariates, which included 19 bioclimactic variables in raster format at the 30 degree second resolution, in addition to an elevation dataset mapped at the same resolution obtained from the *geodata* package [35].

To evaluate an ENM for *Crocidura* species, we performed 5-fold cross validation for each species model to obtain training and testing area under the curve (AUC) data for each iteration of every species modelled [36]. We used the AUC to determine the overall quality and to identify cases where large differences between training and testing AUC indicated issues with overfitting [37]. After confirming that both testing and training AUC values were > 0.7 and consistent with each other, we selected the model with the highest AUC for each species [38]. The selected models were used to project the Relative Occurrence Rate (ROR) on a map of Thailand, using the same 18 bioclimatic variables and elevation cropped to the spatial extent of Thailand [37].

## Results

### Participant demographics and enrollment history

As previously described in Ninwattana, et al. (2025), we collected a total of 351 blood serum samples from study participants [17]. Additionally, oral, nasal, or nasopharyngeal swabs were collected from 234 individuals at four timepoints between 2017 and 2023. After initial enrolment in 2017, individuals were approached for re-enrollment at subsequent collection timepoints. Therefore, data was compiled from participants that enrolled once (n = 157), twice (n = 49), three (n = 17), or four times (n = 11) (Supplemental Table 1, Supplemental Figure 1). The median age at sample collection was 48 years (IQR: 38-59), and sex was equally divided with females representing 50.9% of the study participants (Table 1). Most individuals, 75.6% (n = 177) resided in the village for over 10 years, and half, 50.7% (n = 142) reported primary schooling as their maximum level of education. Over half of the participants reported their primary livelihood involved close contact with animals, including in occupational sectors such as agriculture (14.7%), animal welfare (veterinarian, and park ranger) (8.6%), or extraction industries (mining, logging, guano collection) (30.0%). Non-specific illness was reported in 30.8% (n = 72) of participants occurring within the previous 12 months of sampling. A majority (70.1%, n = 164) of participants reported at least one animal contact within the reporting period, including specific contacts with bats, non-human primates, and rodents.

**Table 1.** Demographic and behavioural characteristics of study participants.

	Count (n = 234)	Percentage
Sex		
Male	115	49.15%
Female	119	50.85%
Age group		
Under 18	8	3.42%
18–35	47	20.09%
36–60	127	54.27%
Above 60	43	18.38%
Residence time period		
Less than 1 year	6	2.56%
1–10 years	51	21.79%
More than 10 years	177	75.64%
Highest education		
Primary	142	50.68%
Secondary	53	22.65%
College	30	12.82%
Primary income		
Agriculture	35	14.96%
Animal welfare	20	8.55%
Extractive industry	70	29.91%
Healthcare worker	7	2.99%
Other	102	43.59%
Illness in previous year	72	30.77%
Animal contacts		
Raised animals	164	70.09%
Scratched or bitten	37	15.81%
Slaughtered animals	17	7.26%
Collected bat guano	82	35.04%
Bat contact	48	20.51%
Handled animal	13	5.56%
Handled waste	34	14.53%
Roost near residence	19	8.12%
Found faeces near food	3	1.28%
Consumed	2	0.85%
Bitten or scratched	4	1.71%
Non-human primate contact	3	1.28%
Worked near	1	0.43%
Handled	2	0.85%
Rodent contact	29	12.39%
Found in residence	18	7.69%
Killed	1	0.43%
Found faeces near food	7	2.99%
Handled	5	2.14%
Bitten or scratched	1	0.43%

### Molecular screening

Over the course of the study, we detected no filovirus and henipavirus nucleic acid in any of the samples. All oral, nasal, or nasopharyngeal swabs from all bat species were negative for nucleic acid evidence of filoviruses and bat-borne paramyxoviruses.

### Serological screening: bats

**Figure 1.**
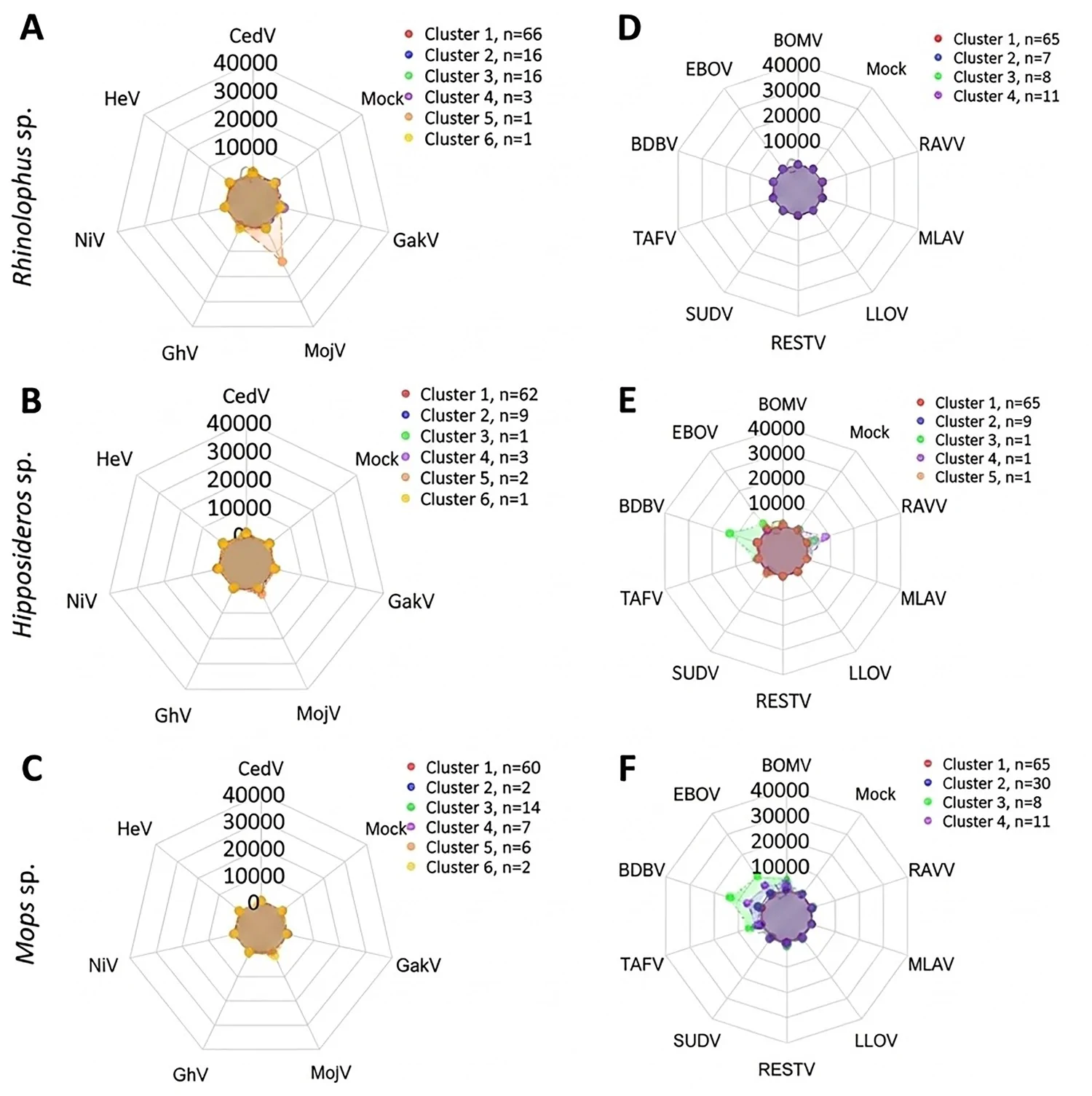
**Serological binding profiles identified anti-orthoebolavirus reactivity in free-tailed and round-leaf bats.** Minimal henipavirus and parahenipavirus seroreactivity was observed among (A) *Rhinolophus* (n = 91), (B) *Hipposideros* (n = 78), and (C) *Mops* (n = 103). No filovirus reactivity was observed within (D) *Rhinolophus* bats, whereas filovirus seroreactivity was observed in (E) *Hipposideros* (Cluster 3, green, and Cluster 4, purple) and (F) Mops (Cluster 3, green). Radial axes represent antigen targets of serological reactivity, and scales as a continuous linear measurement of median ﬂuorescence intensity (MFI). Continuous lines represent the mean MFI within the observed cluster as determined by principal component analysis and k-medoids clustering. CedV, Cedar virus; HeV, Hendra virus; NiV, Nipah virus; GhV, Ghana virus; MojV, Mòjiāng virus; GakV, Gamak virus; BOMV, Bombali virus; EBOV, Ebola virus; BDBV, Bundibugyo virus; TAFV, Taï Forest virus; SUDV, Sudan virus; RESTV, Reston virus; LLOV, Lloviu virus; MLAV, Měnglà virus; RAVV, Ravn virus, Mock; mock protein antigen.

Bats (n = 272) were sampled between 2020 and 2021, including 78 *Hipposideros larvatus*, 103 *Mops plicatus*, 10 *Rhinolophus coelophyllus*, and 81 *R. pusillus* (Supplementary Table 2). In the absence of molecular evidence of filovirus or henipavirus infection, we used multiplex immunoassays to directly measure the infection histories of each individual bat. One *Rhinolophus* bat clustered with reactivity towards MojV (Figure 1A, *cluster* 5); whereas, we detected no seroreactivity with any henipaviruses in the *H. larvatus* and *M. plicatus* bats (Figure 1B-C). Univariate mixture modelling of anti-MojV antibody responses in the *Rhinolophus* population detected a seroprevalence of 2.2% (2/91) (Supplemental Figure 2E-F, Supplemental Figure 3, Supplemental Table 3).

None of the sera samples collected from *Rhinolophus* bats possessed antibodies that reacted with any of the filovirus GP antigens (Figure 1D). Two *Hipposideros larvatus* bats independently clustered into either BDBV/EBOV-reactive or RAVV-reactive groups (Figure 1E, *clusters* 3 and 4, respectively). Mixture models identified threshold cut-offs in both EBOV and BDBV antibody distributions, and seroprevalences for BDBV and EBOV were measured as 9.0% (7/78) and 3.8% (3/78), respectively, including two bats reactive to both viral species (Supplemental Table 3, Supplemental Figures 2B and 4). A strong positive correlation (ρ = 0.96, *p* < 0.001) was observed between EBOV and BDBV reactive samples, implying cross-reactivity (Supplemental Figure 5). Serum samples from eight *Mops plicatus* bats were seroreactive with BOMV, EBOV, BDBV, and TAFV, and two clusters were distinguished by the magnitude of this antibody response (Figure 1F, *clusters* 3 and 4). In *M. plicatus*, we detected seroprevalences of 12.6% (13/103), 9.7% (10/103), 11.6% (12/103), and 6.8% (7/103) for BOMV, EBOV, BDBV, and TAFV, respectively following mixture model analysis, including ten bats reactive with at least two viral species (Supplemental Table 3, Supplemental Figures 2D and 6). We also observed a strong positive correlation with the seroreactive *M. plicatus* and other orthoebolavirus GP antigens, BOMV-EBOV (ρ = 0.72, *p* < 0.001), BOMV-BDBV (ρ = 0.65, *p* < 0.01), BOMV-TAFV (ρ = 0.62, *p* < 0.001), EBOV-BDBV (ρ = 0.92, *p* < 0.0001), EBOV-TAFV (ρ = 0.78, *p* < 0.0001), and BDBV-TAFV (ρ = 0.70, *p* < 0.001) (Supplemental Figure 7), and reactivity with BDBV was significantly higher than other orthoebolaviruses (Supplemental Table 4). These serological profiling patterns strongly imply antisera cross-reactions on the level of the orthoebolavirus genus.

### Serological screening: humans

**Figure 2.**
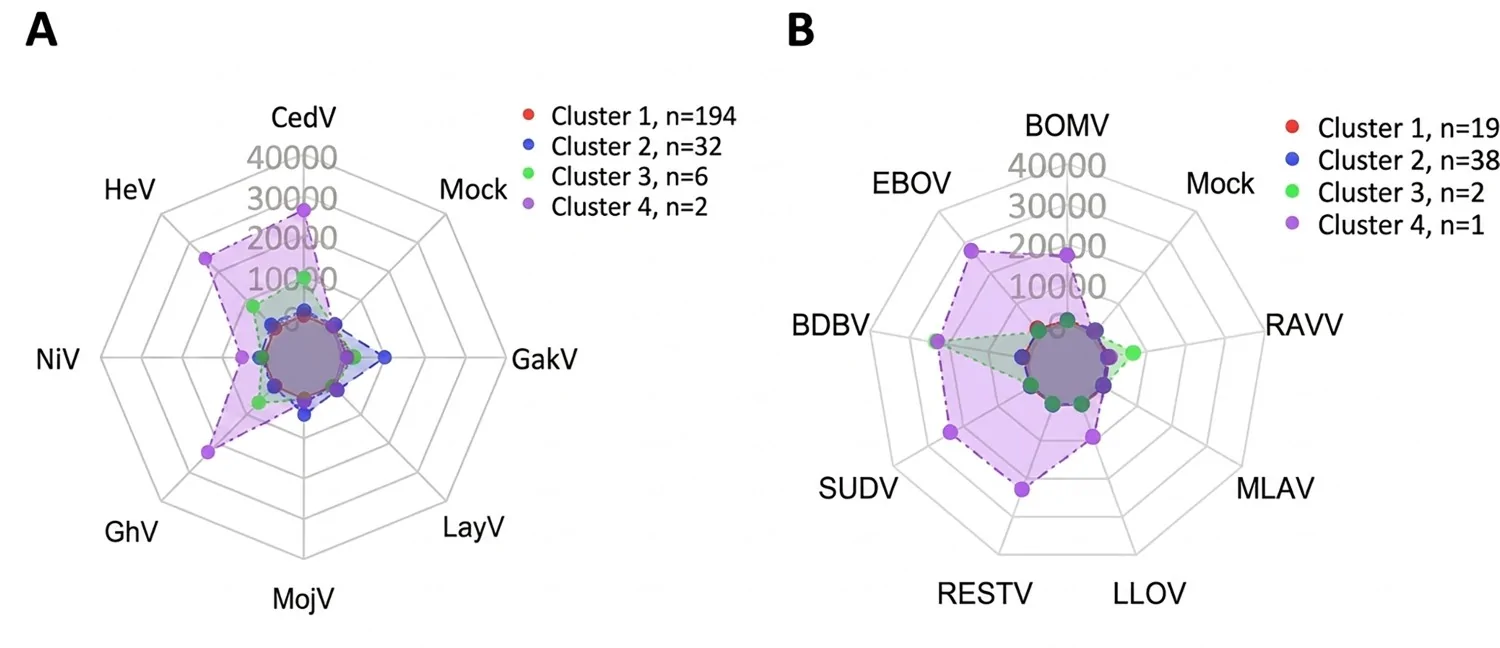
**Detection of parahenipavirus seroreactivity in humans**. (A) Variable reactivity towards bat-borne henipaviruses (Cluster 3, green, and cluster 4, purple) and shrew-borne henipaviruses (Cluster 2, blue) within sampled humans (n = 234). (B) Broad (Cluster 4, purple) and specific (Cluster 3, green) filoviral seroreactivity within the human cohort. Radial axes represent antigen targets of serological reactivity, and scales as a continuous linear measurement of median fluorescence intensity (MFI). Continuous lines represent the mean MFI within the observed cluster as determined by principal component analysis and k-medoids clustering. CedV, Cedar virus; HeV, Hendra virus; NiV, Nipah virus; GhV, Ghana virus; MojV, Mòjiāng virus; GakV, Gamak virus; BOMV, Bombali virus; EBOV, Ebola virus; BDBV, Bundibugyo virus; TAFV, Taï Forest virus; SUDV, Sudan virus; RESTV, Reston virus; LLOV, Lloviu virus; MLAV, Měnglà virus; RAVV, Ravn virus; Mock, mock protein.

We observed distinct seroreactive clusters with the bat-borne henipaviruses, CedV, HeV, NiV, and GhV (Figure 2A, *clusters 3-4*, n = 8), and the terrestrial small mammal-borne parahenipaviruses, GamV, LayV, and MojV (Figure 2A, *cluster 2*, n = 32), in the human serological samples (N = 234), including five samples reactive with two or more viral species. We calculated respective seroprevalences for CedV, HeV, NiV, and GhV of 2.1% (5/234), 2.6% (6/234), 1.3% (3/234), and 1.3% (3/234) (Supplemental Table 3, Supplemental Figures 8A and 9). No individuals seroconverted to a henipavirus over the course of the study. Positive correlation among seroreactive samples was observed between CedV-HeV (ρ = 0.99, *p* < 0.0001), CedV-NiV (ρ = 0.68, *p* < 0.0001), CedV-GhV (ρ = 0.99, *p* < 0.0001), HeV-NiV (ρ = 0.67, *p* < 0.001), HeV-GhV (ρ = 0.99, *p* < 0.0001), and NiV-GhV (ρ = 0.70, *p* < 0.001) (Supplemental Figure 10B).

Seroprevalences for parahenipaviruses in the humans were 1.3% (3/234), 2.6% (6/234), and 6.8% (16/234) for LayV, MojV, and GakV, respectively, including nine samples reactive to two or more viral species (Supplemental Table 3, Supplemental Figure 8B). Four individuals seroconverted to GakV, two seroconverted to MojV, and one seroconverted to both GakV and MojV over the course of the study. Positive correlation was observed among MojV and LayV seroreactivity (ρ = 0.63, *p* < 0.001), whereas no correlation was observed between henipaviruses and parahenipaviruses (Supplemental Figure 10B). Sera from one study participant was co-reactive with all of the orthoebolaviruses (Figure 2B, *cluster 4*), and two serum samples formed a cluster that was defined by reactivity with both BDBV and RAVV (Figure 2B, *cluster 3*). Mixture models did not converge on distinct two or three sub-populations within the antigen–antibody distributions for RAVV GP and no further analysis was performed to determine seroprevalence. For the orthoebolaviruses identified through k-medoids clustering, seroprevalences ranged from 0.4–1.7% for RESTV – BOMV (Supplemental Table 3, Supplemental Figures 8B and 11). One individual seroconverted to both BOMV and EBOV over the course of the study. Positive correlation among reactive samples was observed between BOMV-EBOV (ρ = 0.98, *p* < 0.0001), BOMV-SUDV (ρ = 0.95, *p* < 0.01), BOMV-RESTV (ρ = 0.94, *p* < 0.01), EBOV-SUDV (ρ = 0.90, *p* < 0.05), EBOV-RESTV (ρ = 0.90, *p* < 0.05), and SUDV-RESTV (ρ = 1.00, *p* < 0.0001) (Supplemental Figure 10A).

### Epidemiological analysis

**Figure 3.**
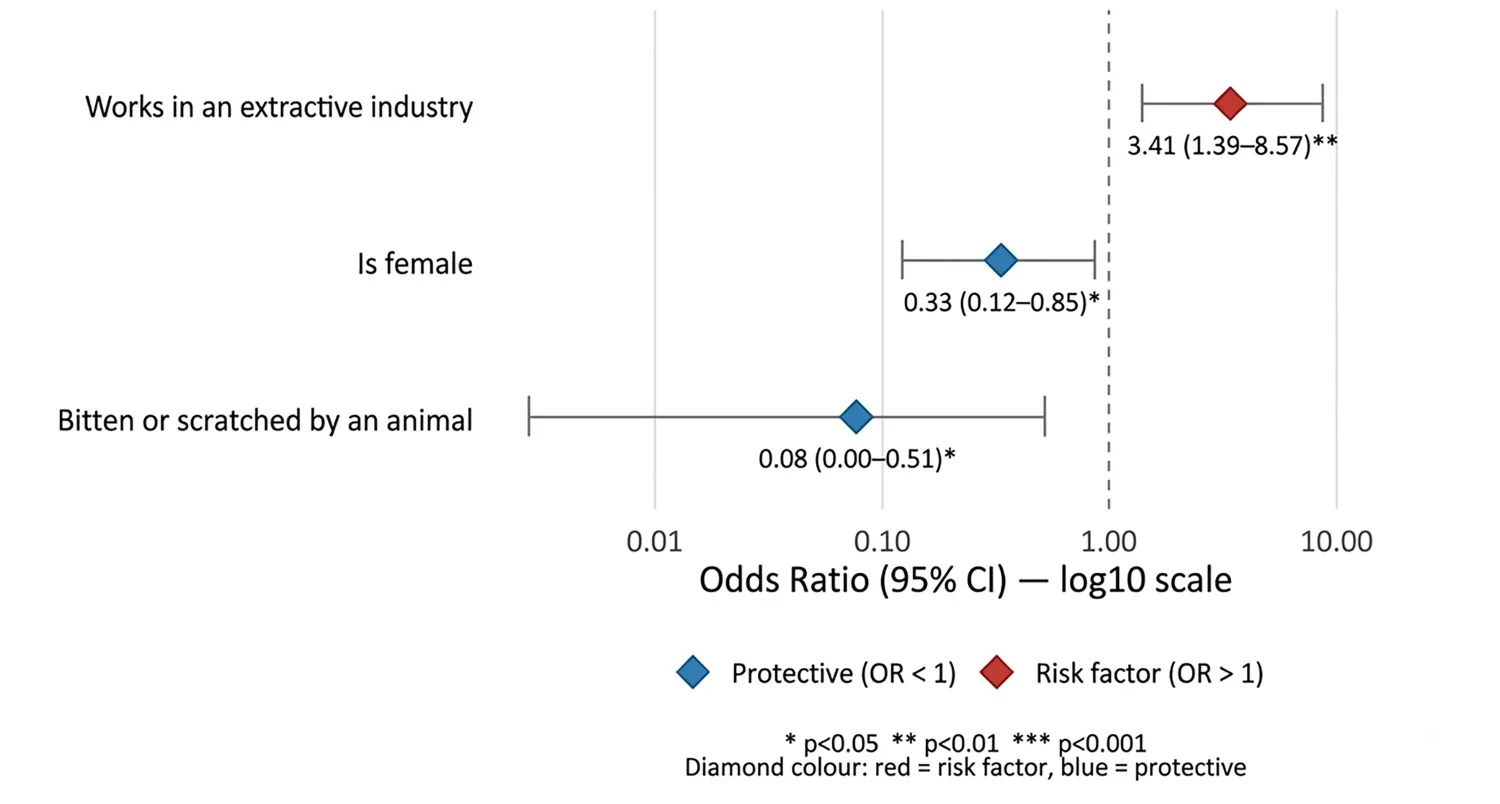
**Reported behaviours reveal associations with parahenipavirus serostatus from post-LASSO logistic regression**. Horizontal axis represents the Odds ratio of a reported variable. Vertical axis describes the predictor variables from post-LASSO regression. A red diamond represents risk factors (Odds Ratio >1), whereas a blue diamond represents protective factors (OR <1). The corresponding Odd’s Ratios and 95% confidence intervals are shown below each point.

Following serostatus designation, LASSO regression with post-selection generalized linear model (GLM) logistic regression and confirmatory debiased LASSO regression, elastic net regression, and stepwise logistic regression to control for bias and assess model power were used to detect associations between an individual’s serostatus and their reported behaviours. No regression model detected any association with either filovirus or bat-borne henipavirus seroreactivity (*p* ≥ 0.94). LASSO regression determined three characteristics significantly associated with terrestrial small mammal-borne parahenipavirus seroreactivity: primary livelihood within the extractive industry (OR = 3.41, CI = 1.39-8.57, *p* < 0.01), being female (OR = 0.33, CI = 0.12-0.85, *p* < 0.05), and being scratched or bitten by an animal (OR = 0.08, CI = 0.00-0.51, *p* < 0.05) were associated with the presence of parahenipavirus seroreactivity (Figure 3). However, subsequent regression modelling to control for LASSO-based regression model bias returned conflicting results. Dibiased regression modelling did not detect any predictor variables that were associated with parahenipavirus serostatus, while elastic net modelling only found working in the extractive industry as significantly associated with a positive parahenipavirus serostatus (OR = 4.46, CI = 1.99-10.4, *p* < 0.01) (Supplemental table 5). Finally, stepwise logistic regression included four predictor variables associated with a positive parahenipavirus serostatus and one variable not associated with but included within the model: working in an extractive industry (OR =   3.84, CI = 1.57-9.31, *p* < 0.01), being female (OR = 0.32, CI = 0.12-0.82, *p* < 0.05), being scratched or pitten by an animal (OR = 0.05, CI = 0.00-0.78, *p* < 0.05), touching a rodent (OR = 32.13, CI = 1.58-650, *p* < 0.05), and reporting rodents in their house (OR = 0.18, CI = 0.01-2.96, *p* > 0.05).

### Ecological niche modelling

**Figure 4.**
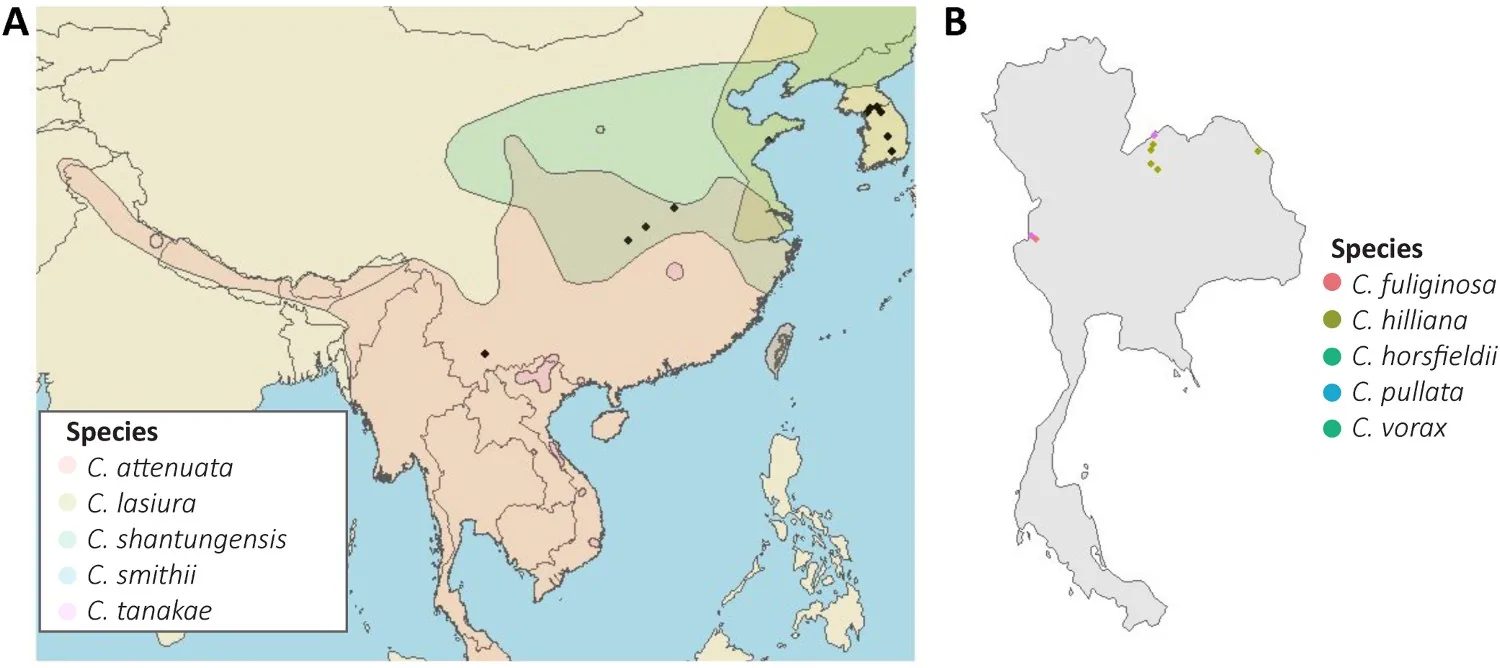
**Distribution of shrew hosts of henipaviruses and *Crocidura* shrews in Thailand. (**A) The distribution of shrew-borne henipaviruses in Asia mapped along with the distribution of host species. B). Shrew occurrences in Thailand obtained from the Global Biodiversity Information Facility (GBIF).

**Figure 5.**
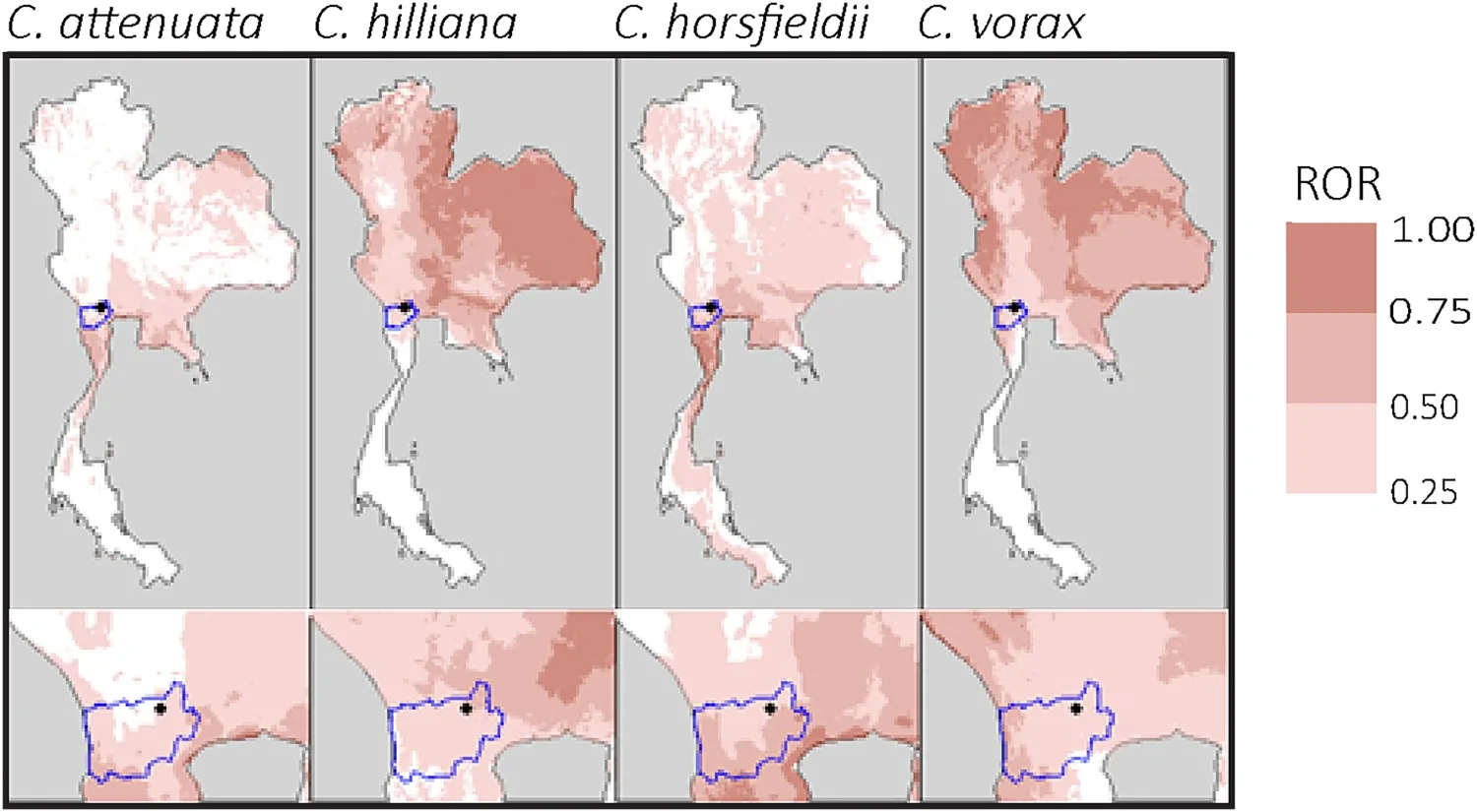
**Projections of MaxEnt models for *Crocidura* shrew species in Thailand**. The Relative Occurrence Ratio is stratified into four groups. The blue outline represents Ratchaburi province, and the point represents the sampling site at Tambon Tao Pun.

Due to the presence of parahenipaviral seroreactivity and the absence of rodent and shrew samples within this study, we constructed a host range map of potential small terrestrial mammal-borne parahenipavirus vectors in Thailand. Of *Crocidura* species previously associated with parahenipaviruses, C*. attenuate* had the broadest distribution covering Southeast Asia (Figure 4A) [39-43]. Based on Global Biodiversity Information Facility data, five *Crocidura* species have been observed in Thailand since 1970: *C. fuliginosa, C. vorax, C. horsfieldii, C. hilliana*, and *C. pullata* (Figure 4B). We calculated the mean projected ROR for each species in Thailand and six species with ROR above 0.05, or having a 5% probability of species presence relative to other geographic locations were identified: *C. horsfieldii*, *C. hilliana*, *C. vorax*, *C. attenuata*, *C. fuligunosa*, and *C. malayna*. Of these, the first four were identified to have a ROR above 0.25 (25% probability of species presence) across Ratchaburi (Figure 5, province outlined in blue, sample site in black). We also mapped the models of *C. lasiura* and *C. shantungensis*, which previously have been identified as henipaviruses hosts, but neither had an ROR above 0.25 across Ratchaburi (Supplemental 12A) [8].

## Discussion

In this study, we conducted serial cross-sections of human study participants with perceived vulnerability to zoonosis through occupational risk, and cave-roosting insectivorous bats at a perceived wildlife-human interface, to detect evidence of exposure to selected WHO priority paramyxovirus and filovirus pathogens within Thailand. Although, we found no genetic evidence of these viruses in either the bats or humans, we did detect serologic evidence of previous exposure to orthoebolaviruses in free-tailed (*Mops sp.*) and roundleaf (*Hipposideros sp.*) bats at the Wat Khao Chong Pran cave site. Our study identified tserologically-reactive antibodies to BOMV, EBOV, BDBV, and TAFV in wrinkle-lipped free-tailed bats (*M. plicatus*). Surprisingly in the human study participants, we detected seroreactivity against three parahenipaviruses, a newly described and expanding genus of paramyxoviruses associated with small terrestrial mammals. These observed serological profiles were primarily driven by reactivity with LayV, MojV, and GakV, suggesting that antigenically-related parahenipaviruses may be distributed in Southeast Asia.

Orthoebolaviruses are associated with free-tailed bats both in other studies and substantiated by the presented findings. The Angolan free-tailed bat (*M. condylurus*) is a host of BOMV in Sierra Leone, Guinea, Southern Kenya, Mozambique, and Tanzania, and has been found in *M. pumilus* in Sierra Leone [44-48]. In Southeast Asia, RESTV RNA has been found in Philippine *M. plicatus* [49]. Experimental infections of *M. condylurus* primary cells with EBOV demonstrated a tolerated and persistent infection further suggesting that species of *Mops* bats may be suitable hosts for orthoebolaviruses [50]. In contrast of the findings within *M. plicatus*, orthoebolavirus seroprevalence in *H. larvatus* was only observed for EBOV and BDBV, and we detected no seroreactivity in the *Rhinolophus* bats. This highlights the importance of studying specific host–pathogen interactions.

These data further suggest an antigenic diversity of Asiatic orthoebolaviruses as evidenced by differences from findings in other studies outside of Thailand. There are presently three species of Asiatic filoviruses described, including RESTV in the Philippines [51], and MLAV and DEHV in *Rousettus* bats in China [11,52-55]. In China, a limited serological study found RESTV-reactive antibodies in multiple species of *Hipposideros* bats and EBOV NP-reactive antibodies in one *H. pomona* [56]. However, in this study we detected no evidence of RESTV in *H. larvatus.* In the absence of additional sera aliquots to perform corroborating neutralization tests with pseudotyped filovirus GP viruses, our data suggest prior exposure to an antigenically-related virus(es) of the orthoebolavirus genus. The lack of seroreactivity observed in rhinolophid species found within the same cave supports conclusions that host–pathogen interactions are specific on the genus-level, and that co-roosting species interactions need to be further studied to understand the potential for inter-species transmission [57].

To date, the only confirmed transmissions of Asiatic filoviruses to humans in Southeast Asia are in the Philippines where RESTV is endemic; outbreaks in pigs and transmission to humans causes subclinical disease [58,59]. There have been no outbreaks or reports of clinically confirmed filoviruses in Thailand. The presented report identified low-levels (0.4-1.7%) of seroprevalence to BOMV, EBOV, BDBV SUDV, and TAFV in human participants. The mixture model threshold cut-offs for BOMV, EBOV, BDBV, SUDV, and RESTV, ranged from 2,772–10,567 MFI (average: 5,781 MFI), consistent with our clinical cohort validated threshold, of anti-EBOV GP IgG cutoff of 8,721 MFI (98% specificity) [23]. Since no seroprevalence exceeded 2%, we concluded that the detected seroreactivity is likely a result of false positives and may be the result of pre-existing human IgG responses against the filovirus GP antigen [23,60,61].

Regarding paramyxoviruses, we detected evidence of previous henipavirus exposure within the human population of our study. All seroreactive samples demonstrated positive correlation with one another, with highest antibody levels against the GhV G antigen. We have previously observed this seroreactivity pattern in humans surveyed in Cambodia and in flying foxes sampled in the Philippines [14,60]. Sequences of an African henipavirus ancestor were previously detected in *Pteropus medius* flying foxes sampled in Bangladesh [14,60,62]. The serologic evidence of a GhV-like henipavirus in human populations may suggest exposure to a related bat-borne paramyxovirus, although the seroprevalences are low, and in the absence of molecular detection in humans or wildlife and assay specificity validation these could also be interpreted as artefacts.

Newly reported seroreactivity in humans in Thailand was observed towards parahenipavirus species, including MojV, LayV, and GamV, which would be expected from the distribution and carrier status of shrews, although supporting evidence to suggest the presence of a parahenipavirus in the human and wildlife community is lacking. Critical for public health initiatives, behavioural analysis in three of the four models used within this population found extractive industry employment positively associated with parahenipavirus seroreactivity. Debiased LASSO regression did not determine any predictor variables associated with parahenipavirus serostatus. This may arise from a weak association between the predictor variables and serostatus or a lack of statistical power within the given dataset resulting from low overall sample numbers or the oversampling of individuals employed within the extractive industry. Parahenipaviruses have been primarily detected in shrews (genus *Crocidura*) across five continents, and there is broad host distribution and overlap in East and Southeast Asia (Supplemental Figure 12B). MojV was first discovered in rodents following the investigation of the deaths of three cave miners in Southern China [63], and investigations following an outbreak of acute febrile illness in Eastern China implicated shrews as a host species for LayV [8]. Two additional parahenipaviruses, GamV and DarV, were found in South Korea in *C. lasiura* and *C. shantungensis*, and *in-vitro* investigations have demonstrated GamV can infect human lung epithelial cells [9].

By utilizing MaxEnt modelling, we were able to produce maps projecting which *Crocidura* species are likely to inhabit proximally to our sample site. Of the 44 Asian *Crocidura* species for which we produced projections, three had ROR greater than 0.25 across all of Ratchaburi province. Of these species (*C. hilliana, C. vorax,* and *C. attenuata),* only *C. attenuata* has previously been implicated as a host of a parahenipavirus [64]. However, parahenipaviruses have been detected in species of a single genus, so it is possible that the other two species could potentially be hosts [8] (Supplemental Figure 12C). MaxEnt models, especially those produced solely using presence data, are imperfect measures of species presence, but in the absence of ecological sampling they identified species that may be part of the zoonotic interface for parahenipaviruses.

The presented research is predominantly limited by sample size. Furthermore, a lack of additional sera and appropriate pseudoviral neutralization platforms precluded secondary testing to characterize sera neutralizing activity and findings are therefore not corroborated by neutralizing activity. Additionally, given the strong correlation observed among viral species within the tested viral genera, species-specific conclusions regarding viral exposure cannot be concluded. Therefore, follow up projects including consistent sampling with multiple timepoints across a broader population and appropriate sera for neutralization tests would be necessary for a deeper understanding of viral spillover and transmission among the human population. Where possible, bootstrapping was used to reduce the impact of low sample size, but in follow up projects, a greater sample size would provide greater stability in odds ratios and confidence interval calculations. Additionally, no small terrestrial mammals were included in this study and no conclusions regarding the potential host species responsible for the spillover of viruses responsible for the observed paraheipaviral seroprevalence in humans can be made. A more thorough sampling of the bat population and expanding sampling to include rodents and shrews within the area is essential for understanding the potential host-populations of paramyxoviruses within the region, however there are limited sustainable wildlife surveillance systems within the country and a concentrated effort between public health and wildlife authorities to adequately address zoonotic disease surveillance at relevant interfaces would be required. Finally, although MaxEnt modelling is generally robust with small sample sizes, several species modelled had exceptionally small sample sizes. Further ecological sampling therefore presents an opportunity to both better understand the prevalence of viruses in these shrews and potential interfaces.

This study presents evidence of exposure to previously undetected filoviruses and paramyxoviruses in Thailand and lays the framework for further investigations into behaviours associated with zoonosis. The relevance of the serological findings within this study as they relate to human health remain elusive, as there was no confirmed link to active disease nor sustained viral presence within the country. Future biosurveillance efforts should expand on the findings of this study and broaden the animal species sampled to better understand the zoonotic interfaces within the country.

## Supplementary Material

Supplemental Material

## Data Availability

Data and key materials utilized in this study are available as a sharable resource upon reasonable request to the corresponding authors.

## Abbreviations

Nipah virus (NiV), Hendra virus (HeV), Cedar virus (CedV), Ghana virus (GhV), Langya virus (LayV), Mòjiāng virus (MojV), Gamak virus (GakV); Ebola virus (EBOV), Bundibugyo virus (BDBV), Bombali virus (BOMV), Sudan virus (SUDV), Taï Forest virus (TAFV), Reston virus (RESTV), Ravn virus (RAVV), Měnglà virus (MLAV) tetrameric receptor-binding glycoproteins (G), trimeric native-like attachment glycoprotein ectodomains (GP). Polymerase chain reaction (PCR), ecological niche modelling (ENM), least absolute shrinkage and selection operator (LASSO), generalized linear models (GLM).

## Authors’ information

SLS, NM, BG, and MDR are employees of the Henry M. Jackson Foundation for the Advancement of Military Medicine. SLS, NM, BG, and MDR are employees of the Henry M. Jackson Foundation for the Advancement of Military Medicine. EDL is a federal employee and this work was prepared as part of official duties. The opinions and assertions expressed herein are those of the author(s) and do not reflect the official policy or position of the Henry M. Jackson Foundation for the Advancement of Military Medicine, Uniformed Services University of the Health Sciences or the Department of War.
