# Supplementary material for: Surveillance for Asiatic filoviruses and zoonotic paramyxoviruses at a perceived wildlife–human interface
Source: Emerg Microbes Infect. 2026 Aug 27;15(1):2713324. doi: 10.1080/22221751.2026.2713324 (PMC13523113; doi:10.1080/22221751.2026.2713324)
Supplement: Supplementary file 1 [file TEMI_A_2713324_SM4746.docx]

**Supplementary Appendix**

**Surveillance for Asiatic filoviruses and zoonotic paramyxoviruses at a perceived wildlife-human interface**

Spencer L. Sterling^a,b,c,d^, Neil Mittal ^b,d^, Benjamin Greene ^b,d^, Khwankamon Rattanatumhi^c^, Sasiprapa Ninwattana^c^, McKenna D. Roe^b,d^, Patarapol Maneeorn^e^, Prateep Duengkae^f^, Supaporn Wacharapluesadee^c^*, Eric D. Laing ^b^*, Opass Putcharoen^c^

*^a^International Graduate Program in Medical Sciences, Faculty of Medicine, Chulalongkorn University, Bangkok, Thailand; ^b^Department of Microbiology and Immunology, Uniformed Services University, Bethesda, MD, USA; ^c^Thai Red Cross Emerging Infectious Diseases Clinical Center, King Chulalongkorn Memorial Hospital, Bangkok, Thailand; ^d^Henry Jackson Foundation for the Advancement of Military Medicine, Bethesda, MD, USA; ^e^Department of National Parks, Wildlife and Plant Conservation, Ministry of Natural Resources and Environment, Bangkok, Thailand; ^f^Department of Forest Biology, Faculty of Forestry, Kasetsart University, Bangkok, Thailand*

**Supplemental Table 1. Participant enrollment by year and number**

| **Number of Enrollments** | **Number of Participants** | **Year** | | | |
| --- | --- | --- | --- | --- | --- |
|  |  | 2017 | 2018 | 2021 | 2023 |
| Single | 157 | 63 | 63 | 8 | 23 |
| Double | 49 | 26 | 25 | 20 | 27 |
| Triple | 17 | 11 | 11 | 17 | 12 |
| Quadruple | 11 | 11 | 11 | 11 | 11 |
| **Total** | 234 | 111 | 110 | 56 | 73 |

**Supplemental Table 2. Bat sample collection by year and species**

| **Genera** | **Species** | **Total Number** | **Year** | |
| --- | --- | --- | --- | --- |
|  |  |  | *2020* | *2021* |
| *Hipposideros* | *larvatus* | 78 | 28 | 50 |
| *Mops* | *plicatus* | 103 | 72 | 31 |
| *Rhinolophus* | *coelophyllus* | 10 | 0 | 10 |
|  | *pusillus* | 81 | 0 | 81 |
| **Total** |  | 272 | 100 | 172 |

**Supplemental Table 3. Cutoffs for human and bats for each tested antigen**

| **Population** | **Virus** | **2 population seropositivity cutoff (MFI, % specificity)** | **Seropositive (N)** | **Seroprevalence (%)** |
| --- | --- | --- | --- | --- |
| Humans | CedV | 8912 (100) | 5 | 2.1 |
|  | HeV | 7773 (100) | 6 | 2.6 |
|  | NiV | 6958 (100) | 3 | 1.3 |
|  | GhV | 8417 (96) | 3 | 1.3 |
|  | MojV | 6842 (95) | 6 | 2.6 |
|  | LayV | 5769 (94) | 3 | 1.3 |
|  | GakV | 9263 (96) | 16 | 6.8 |
|  | BOMV | 3706 (91) | 4 | 1.7 |
|  | EBOV | 8152 (100) | 3 | 1.3 |
|  | BDBV | 10567 (100) | 3 | 1.3 |
|  | SUDV | 2772 (90) | 2 | 0.8 |
|  | RESTV | 3710 (91) | 1 | 0.4 |
| *Hipposideros* spp. | EBOV | 988 (84) | 3 | 3.8 |
|  | BDBV | 872 (93) | 7 | 9.0 |
| *Mops* spp. | BOMV | 1964 (97) | 13 | 12.6 |
|  | EBOV | 2526 (99) | 10 | 9.7 |
|  | BDBV | 4125 (92) | 12 | 11.6 |
|  | TAFV | 2310 (99) | 7 | 6.8 |
| *Rhinolophus* spp. | MojV | 1045 (100) | 2 | 2.2 |

**Supplemental Table 4. Mean difference and Tukey’s Multiple Comparisons Test among seroreactive *M. plicatus* samples**

|  | BOMV | EBOV | BDBV |
| --- | --- | --- | --- |
| EBOV | -1010 (ns) |  |  |
| BDBV | -3679 (*p*=0.0008) | -2669 (*p*=0.0235) |  |
| TAFV | -515.3 (ns) | 494.9 (ns) | 3164 (*p*=0.0049) |

**Supplemental Table 5. Comparison between multiple regression methods to determine behavioral associations with parahenipavirus seroreactivities in humans.**

|  | **Regression Model** | | | | | | | |
| --- | --- | --- | --- | --- | --- | --- | --- | --- |
| **Predictor Variable** | Lasso with post-selection GLM | | Debiased LASSO | | Elastic Net | | Stepwise logistic regression | |
|  | OR  (95% CI) | p-value | OR  (95% CI) | p-value | OR  (95% CI) | p-value | OR  (95% CI) | p-value |
| Extractive Industry | 3.41 (1.39-8.57) | <0.01 | - | - | 4.46 (1.99-10.40) | <0.001 | 3.84 (1.57-9.41) | p<0.01 |
| Female | 0.33 (0.12-0.85) | <0.05 | - | - | - | - | 0.32 (0.12-0.82) | p<0.05 |
| People in dwelling | 0.87 (0.68-1.09) | ns | - | - | - | - | - | - |
| Farming | unstable | ns | - | - | - | - | - | - |
| Scratched or bitten by an animal | 0.08 (0.00-.0.51) | <0.05 | - | - | - | - | 0.05 (0.00-0.78) | p<0.05 |
| Touched a rodent | unstable | ns | - | - | - | - | 32.13  (1.58 - 650) | p<0.05 |
| Handled a bat | unstable | ns | - | - | - | - | - | - |
| Rodent in house | - | - | - | - | - | - | 0.18 (0.01-2.96) | ns |
| (-) indicates variable exclusion from the corresponding model  ns = not significant | | | | | | | | |

**Supplemental Figure 1. STROBE chart demonstrating human enrollment and re-enrollment.** A total of 350 samples and corresponding epidemiological data were collected from 234 individuals between 2017 and 2023.

**Supplemental Figure 2. Connected dot plots demonstrating individual IgG seroreactivity from individual bat genera towards target henipavirus and filovirus glycoproteins.** (A) *Hipposideros* henipavirus, (B) *Hipposideros* filovirus, (C) *Mops* henipavirus, (D) *Mops* filovirus, (E) *Rhinolophus* henipavirus, (F) *Rhinolophus* filovirus. Each column of the horizontal axis represents the target glycoprotein, whereas the vertical axis represents the observed median fluorescence intensity (MFI) of IgG binding towards the target antigen. Grey dashed lines between datapoints connect values observed within given individual samples, the horizontal red dashed lines for filovirus reactivity represent the qualified human seroreactivity threshold previously established in West African Ebolavirus survivors, and the blue dashed lines represent the order- and antigen-specific reactivity thresholds.

**Supplemental Figure 3. Mixture model analysis of *Rhinolophus* spp. binding to the MojV RBP (G).** Horizontal axis represents the log-transformed median fluorescence intensity for each viral antigen, and the vertical axis represents the density of individuals within each horizontal bin. The left plot represents mixture model analysis set to two populations, whereas the right plot represents mixture model analysis set to three populations. Colored lines on each plot represent the proposed distributions of each population within the model.

**Supplemental Figure 4. Mixture model analysis of *Hipposideros larvatus* filovirus binding for each viral antigen.** (A) EBOV and (B) BDBV. Horizontal axis represents the log-transformed median fluorescence intensity for each viral antigen, and the vertical axis represents the density of individuals within each horizontal bin. The left column represents mixture model analysis set to two populations, whereas the right column represents mixture model analysis set to three populations. Colored lines on each plot represent the proposed distributions of each population within the model.

**Supplemental Figure 5. Correlation matrix of filovirus reactive *Hipposideros* samples (n=8).** Intensity and significance of the correlation is shown in the upper-right, whereas the Pearson’s Correlation Coefficient is shown on the bottom-left. Positive correlation (*r*=0.96) was observed between EBOV- and BDBV-reactive samples at the *p*<0.001 level. EBOV, Ebola virus; BDBV, Bundibugyo virus

**Supplemental Figure 6. Mixture model analysis of *Mops plicatus* filovirus binding for each viral antigen.** (A) BOMV, (B) EBOV, (C) BDBV, and (D) TAFV. Horizontal axis represents the log-transformed median fluorescence intensity for each viral antigen, and the vertical axis represents the density of individuals within each horizontal bin. The left column represents mixture model analysis set to two populations, whereas the right column represents mixture model analysis set to three populations. Colored lines on each plot represent the proposed distributions of each population within the model.

**Supplemental Figure 7. Correlation matrix of filovirus reactive *Mops* samples (n=20).** Intensity and significance of the correlation is shown in the upper-right quadrants, whereas the Pearson’s Correlation Coefficients are shown on the bottom-left quadrants. Significant positive correlation (**=*p*<0.01, ***=*p*<0.001, ****=*p*<0.0001) was observed for all combinations of BOMV, EBOV, BDBV, and TAFV reactivity.

**Supplemental Figure 8. Connected dot plots demonstrating individual IgG seroreactivity in humans towards (A) henipavirus and (B) filovirus glycoproteins.** Each column of the horizontal axis represents the target viral glycoprotein, whereas the vertical axis represents the observed median fluorescence intensity (MFI) of IgG binding towards the target antigen. Grey dashed lines between datapoints connect values observed within given individual samples, the horizontal red dashed lines for filovirus reactivity represent the qualified human seroreactivity threshold previously established in West African Ebolavirus survivors, and the blue dashed lines represent the order- and antigen-specific reactivity thresholds.

**Supplemental Figure 9. Mixture model analysis of human henipavirus binding for each viral antigen.** (A) CedV, (B) HeV, (C) NiV, (D) GhV, (E) MojV, (F) LayV, and (G) GakV. Horizontal axis represent the log-transformed median fluorescence intensity for each viral antigen, and the vertical axis represents the density of individuals within each horizontal bin. The left column represents mixture model analysis set to two populations, whereas the right column represents mixture model analysis set to three populations. Colored lines on each plot represent the proposed distributions of each population within the model.

**Supplemental Figure 10. Correlation matrix of (A) filovirus and (B) henipavirus reactive human samples (n=6 and 27, respectively).** Intensity and significance of the correlation is shown in the upper-right quadrants, whereas the Pearson’s Correlation Coefficients are shown on the bottom-left quadrants. Significant positive correlation was observed between BOMV-EBOV (*p*<0.0001), BOMV-SUDV (*p*<0.01), BOMV-RESTV (*p*<0.01), EBOV-SUDV (*p*<0.05), EBOV-RESTV (*p*<0.05), and SUDV-RESTV (*p*<0.0001), between CedV-HeV (*p*<0.0001), CedV-HeV (*p*<0.0001), CedV-GhV (*p*<0.0001), HeV-NiV (*p*<0.001), HeV-GhV (*p*<0.0001), and NiV-GhV (*p*<0.0001), and between MojV-LayV (*p*<0.001).

**Supplemental Figure 11. Mixture model analysis of human filovirus binding for each viral antigen.** (A) BOMV, (B) EBOV, (C) BDBV, (D) SUDV, (E) RESTV. Horizontal axis represent the log-transformed median fluorescence intensity for each viral antigen, and the vertical axis represents the density of individuals within each horizontal bin. The left column represents mixture model analysis set to two populations, whereas the right column represents mixture model analysis set to three populations. Colored lines on each plot represent the proposed distributions of each population within the model.

**Supplemental Figure 12. Distribution and phylogenetic relationships of parahenipaviruses with shrew and rodent host species.** A) Relative Occurrence Rates (ROR) of five additional shrew species in Thailand. Bioclimatic and elevation data associated with habitats of five members of the Crocidura genus were plotted on a map of Thailand to predict habitable regions of each species. *C. fuliginosa, C. horsfieldii, C. lasiura, C. malauna,* and *C. shantungensis* were not predicted to inhabit Ratchaburi province. B) The phylogeny of parahenipavirus (with NiV, (Nipah Virus) serving as the outgroup for the rooted tree) reveals that the parahenipaviruses utilize diverse mammalian hosts. C) Parahenipaviruses borne by small mammals are widely distributed geographically across multiple continents and in multiple countries.
